# Analysis of detergent-free lipid rafts isolated from CD4^+ ^T cell line: interaction with antigen presenting cells promotes coalescing of lipid rafts

**DOI:** 10.1186/1478-811X-9-31

**Published:** 2011-12-08

**Authors:** Colleen Kennedy, Matthew D Nelson, Anil K Bamezai

**Affiliations:** 1 Department of Biology, Villanova University, 800 Lancaster Avenue, Villanova, PA 19085, USA; 2 Current Address: Physician Assistant Program, Cornell Medical College, 1300 York Avenue, New York, NY 10065, USA; 3 Current Address: Department of Neurology, University of Pennsylvania, 415 Curie Blvd., Philadelphia, PA 19104, USA

**Keywords:** raft coalescence, CD4^+ ^T cells, antigen presenting cells, electron microscopy, raft-ELISA

## Abstract

**Background:**

Lipid rafts present on the plasma membrane play an important role in spatiotemporal regulation of cell signaling. Physical and chemical characterization of lipid raft size and assessment of their composition before, and after cell stimulation will aid in developing a clear understanding of their regulatory role in cell signaling. We have used visual and biochemical methods and approaches for examining individual and lipid raft sub-populations isolated from a mouse CD4^+ ^T cell line in the absence of detergents.

**Results:**

Detergent-free rafts were analyzed before and after their interaction with antigen presenting cells. We provide evidence that the average diameter of lipid rafts isolated from un-stimulated T cells, in the absence of detergents, is less than 100 nm. Lipid rafts on CD4^+ ^T cell membranes coalesce to form larger structures, after interacting with antigen presenting cells even in the absence of a foreign antigen.

**Conclusions:**

Findings presented here indicate that lipid raft coalescence occurs during cellular interactions prior to sensing a foreign antigen.

## Background

Signals emanating from the plasma membrane have spatial and temporal components [1-5]. Spatial distribution and accessibility of signaling proteins on the plasma membrane can potentially have profound effects on the outcome of signaling. While knowledge of temporal signaling events has rapidly advanced, the spatial distribution of signaling proteins remains unclear. More so, how the spatial distribution of signaling molecules relates to temporal signaling is unknown. However, recently, re-organization on the plasma membrane of quiescent cells was recognized after triggering signaling from the membrane [6-11].

Lipid raft membrane domains are rich in cholesterol and sphingolipids and known to compartmentalize signaling proteins [12-17]. Heterogeneity of lipid rafts, with respect to protein composition, on the plasma membrane may provide an additional level of spatial segregation [18-26]. Ligand and receptor induced molecular interactions on the plasma membrane trigger a signaling cascade that culminates into specific gene expression. Compositional heterogeneity of lipid rafts on the surface of quiescent cells and their subsequent coalescence, when the receptors engage their ligands, might promote interactions between appropriate signaling proteins [14,27]. However, this is only one of several proposed models to explain signal transduction from the plasma membrane to the interior of the cell [28-35].

Lipid rafts assemble to form an immunological synapse, a central structure at the contact site of CD4^+ ^T cells and antigen presenting cells involved in regulating cell signaling [36-45]. These early signaling events are crucial in generating a response by T cells, especially since CD4^+ ^T cells are capable of generating specific cellular responses after the engagement of the same antigen receptor, ranging from differentiation to Th1 or Th2 or Th17 (T helper cell subsets).

In light of the observation that lipid rafts are compositionally heterogeneous, it remains unclear whether distinct sub-populations of rafts assemble at or around the synapse and thus, contribute to signal transduction and distinct cellular responses. Methods allowing enumeration of lipid rafts as on a single raft and sub-population basis in quiescent, activated, and differentiating cells will aid in addressing the role of lipid rafts in signaling. To enumerate lipid rafts in T cells, we have used a published detergent-free isolation procedure [46]. Lipid rafts isolated from a T cell line in the presence and absence of a specific antigen were visualized by transmission electron microscopy. It was surprising to find that lipid rafts isolated from co-cultures of CD4^+ ^T cell and antigen presenting cells in the absence of antigen show raft coalescence/clustering.

## Materials and methods

### Cell Culture

Mouse CD4^+ ^T-T hybrid of Th1 phenotype YH16.33 [47] and A20 [48] cell lines (generous gifts from Dr. Ken Rock, University of Massachusetts Medical Ctr, MA) were grown in Dulbecco's modified eagle medium (DMEM) with 4.5 g/ml of glucose (Invitrogen, Carlsbad, CA) supplemented with 10% heat inactivated fetal bovine serum, L-glutamine (Atlanta Biologicals, Atlanta, GA), sodium pyruvate, penicillin/streptomycin, and fungizone (Invitrogen, Carlsbad, CA). Cell cultures were maintained at 37°C in a 10% CO_2 _incubator.

### Detergent-Free Isolation Protocol

Lipid rafts were isolated using a previously published protocol [46]. Briefly, 6 × 10^7 ^of total cells either YH16.33 alone or co-cultured with A20 (1:1 ratio) in the presence or absence of 1 mg/ml chicken ovalbumin (antigen) was cultured for 16-18 hrs. Cells were centrifuged for 5 minutes at 1000 × g at 4°C. The supernatant was decanted; the pellet was re-suspended in 10 ml of base buffer solution consisting of 20 mM Tris-HCl, 250 mM Sucrose (pH 7.8), supplemented with 1 mM CaCl_2 _and 1 mM MgCl_2 _followed by centrifugation for 2 minutes at 250 × g at 4°C. Then the supernatant was decanted, the pellet was re-suspended in 1 ml of the base buffer solution supplemented with, CaCl_2 _and MgCl_2_, a protease inhibitor cocktail set (EMD BioSciences, Darmstadt, Germany), and a calpain inhibitor (Sigma-Aldrich, St. Louis, MO), and then lysed by passaging through a ¾ inch 23 gauge needle, 20 times. The lysate was centrifuged at 1000 × g for 10 minutes at 4°C. The supernatant was collected and stored on ice. The pellet was re-suspended with 1 ml of the base buffer solution supplemented with CaCl_2_, MgCl_2_, and protease inhibitor and lysed again by passaging through a ¾ inch 23 gauge needle, 20 times. The lysate was centrifuged at 1000 × g for 10 minutes at 4°C. The supernatant was pooled with the previously collected supernatant. Two ml of the base buffer supplemented with an equal volume of 50% Optiprep solution (Sigma Aldrich, St. Louis, MO) was transferred to an ultracentrifuge tube (Beckman Instruments, Paolo Alto, CA). The solution was then overlaid with 1.6 ml each of 20%, 15%, 10%, 5% and 0% Optiprep solution, respectively, with a total final volume of 12 ml. The gradient was centrifuged for 90 minutes at 52,000 × g at 4°C in an ultracentrifuge (Beckman Instruments, Paolo Alto, CA). The sample was then fractionated in 1.3 ml aliquot from the top of the gradient and stored at -20°C. For detergent isolation experiments, lipid rafts were obtained in the presence of 1% Triton X-100 and subjected to sucrose density gradient as described previously [23,49].

### Western Blot Analysis

Fifteen μl of each fraction was combined with 6.3 μl of lithium dodecyl sulfate (LDS) buffer (Invitrogen, Carlsbad, CA) and 2.3 μl DTT (Invitrogen, Carlsbad, CA). Twenty-two μl of the fraction solution was loaded into 4-15% gels (BioRad, Hercules, CA). The gel was electrophoresed using 2-(N-morpholino) ethanesulfonic acid (MES) buffer (Invitrogen, Carlsbad, CA) at 100 volts for approximately 45 minutes. The gel was then transferred to a polyvinylidene fluoride (PVDF) membrane for 1 hour at 45 volts. The membrane was blocked with 5% non-fat Carnation Instant milk prepared in phosphate buffer saline solution with Tween-20 (PBST) (Sigma Aldrich, St. Louis, MO) and incubated with appropriate primary antibodies against Linker of Activated T cells (LAT), β-COP (Santa Cruz Biotechnology Inc, CA), overnight at 4°C. The species specific, secondary antibodies conjugated to horseradish peroxidase (HRP) (Pierce, Rockford, IL) were added and incubated for 75 minutes at room temperature. The membrane was then exposed to substrate and chromogen solution, a mixture of equal volumes of H_2_O_2 _and a luminol solution (SuperSignal West Dura) (Pierce, Rockford, IL) for 2 minutes and then exposed using an image analyzer (Alpha-Innotech, San Leandro, CA).

### Dot Blot Protocol

PVDF membranes were soaked in methanol for two minutes to moisten the membrane. Three μl dots of fraction samples were placed on the PVDF membrane. The samples were allowed to dry on the membrane, and blocked with 5% non-fat Carnation Instant milk prepared in PBST for 60 minutes at room temperature. The membrane was then incubated in cholera toxin β chain conjugated to HRP (BD Biosciences, San Jose, CA) for 60 minutes. The membrane was then exposed to SuperSignal West Dura (Pierce, Rockford, IL) substrate for 2 minutes and then exposed using an image analyzer (Alpha-Innotech, San Leandro, CA).

### Raft ELISA Protocol

Lipid rafts were analyzed by raft-ELISA as reported in previous publications [23,49], with one exception: detergent-free rafts were used instead of the detergent-resistant rafts. Briefly, 96 well flat bottom, high bonding, enzyme immuno-assay/radioimmuno assay (EIA/RIA) plates (Costar, New York, NY) were coated with 50 μl capture antibody (2 μg/ml) and covered with saran wrap and incubated at 4°C overnight. The microwells were then washed with 100 μl of wash buffer, PBST, 4 times. Wells were then blocked with blocking buffer PBST supplemented with 1% (w/v) fraction V bovine serum albumin (BSA) (PBST/BSA), (Fisher Scientific, Pittsburg, PA) for 30 minutes at room temperature. Excess of blocking reagents were removed with washing buffer, PBST; this step was repeated three times. Fifty μl samples (1:5 diluted raft fractions in PBST/BSA) were added to wells and incubated overnight at 4°C. Unbound lipid rafts were removed by washing with PBST 9 times. Biotinylated detection antibody (1 μg/ml) was added to each microtitre well and incubated for 1 hour at room temperature followed by washing unbound antibody 6 times with PBST. Avidin-HRP was added to each well and incubated for 30 minutes at room temperature. Unbound avidin-HRP conjugate was removed by washing 8 times with PBST. A 100 μl solution of a 1:1 mixture of 2,2'-azino-di[3-ethyl-benzthiazoline 6-sulphonate] (solution A) and 0.02% solution of H_2_O in citric acid buffer (solution B) were added to appropriate well. The absorbance was read at 405 nm with a Spectramax 190 plate reader (Molecular Devices, Sunnyvale, CA).

### Formvar Coating EM Grids

Coating of nickel grids with formvar was carried out according to previous publications. Nickel grids (Electron Microscopy Sciences, Fort Washington, PA) were sonicated 3 times in ethanol prior to their use. Clean microscopic glass slides were dipped into a formvar solution in ethylene dichloride (Electron Microscopy Sciences, Fort Washington, PA) and chloroform (Fisher Scientific, Pittsburg, PA) for a few seconds to allow coating of formvar on the slide. The edges of the glass slides were scored and tilted to release the formvar in a clean bowl of double distilled water. Nickel grids were mounted on top of the floating formvar sheets. Using a different microscope slide wrapped in parafilm, the floating formvar, with the grids on top, was carefully scooped up from the water bowl and allowed time to dry and store at RT until further use.

### Immunogold labeling for TEM

Lipid rafts were captured and detected by the method we have previously used for detection of detergent isolated lipid rafts [23,49]. A capture antibody, purified anti-mouse CD90 (Thy-1) (G7) (BD Biosciences, San Jose, CA) was coated on the nickel grid at 4 μg/ml antibody concentration in carbonate/bicarbonate buffer in a humid chamber. Antibody coating was carried out by placing the formvar coated side of the grid faces down on a drop of carbonate-bicarbonate buffer with capture antibody for an overnight period at 4°C. Nickel grids were washed 4 times with phosphate buffer saline (13.7 mM NaCl, 0.27 mM KCl, 0.43 mM Na_2_HPO4-7H_2_0, 0.14 mM KH_2_PO4, pH 7.3) supplemented with 1% BSA-C (Aurion, Costerweg, Netherlands). For each washing step, grids were incubated with the washing buffer for 5 minutes at room temperature in a humid chamber. Non-specific sites on the grids were then blocked with a blocking buffer consisting of 1 × PBS supplemented with 0.05% (w/v) of fraction V bovine serum albumin for 20 minutes at room temperature. Grids were then washed with incubation buffer 2 times, 5 minutes each, at room temperature followed by incubation with 30 μl drops of lipid raft fraction samples for an overnight period at 4°C. Unbound lipid rafts were removed by washing with PBS/BSA buffer at room temperature, and this step was repeated 5 times. Grids were than incubated with biotin-conjugated detection antibody Ly6A/E (Sca-1) (D7) (BD Biosciences, San Jose, CA) at 3 μg/ml in PBS-BSA buffer for 60 minutes at room temperature. Grids were washed 4 times with PBS-BSA buffer at room temperature to remove excess detection antibody. Non-specific sites in the grids were blocked by incubating on top of 30 μl droplets of blocking buffer for 15 minutes at room temperature followed by incubation with goat anti-biotin antibody conjugated to 10 nm gold particles at a 1:250 dilution of the stock (Aurion, Costerweg, Netherlands) for 60 minutes at room temperature. Grids were washed 2 times with double distilled water (ddw) for 5 minutes each at room temperature and incubated on 30 μl drops of 1% gluteraldehyde (Electron Microscopy Sciences, Fort Washington, PA) in double distilled water for 5 minutes at room temperature. Grids were allowed time to dry, preparation side up, on Whatmann paper after washing with ddw. Lipid rafts on the grid were fixed with 1% osmium tetroxide (Electron Microscopy Sciences, Fort Washington, PA) in double distilled water for 10 minutes. This process was followed by counter staining with1% tannic acid (Electron Microscopy Sciences, Fort Washington, PA) and 2% uranyl acetate (Electron Microscopy Sciences, Fort Washington, PA) in double distilled water for 30 minutes at room temperature, under a cover to prevent light exposure. Grids were washed with double-distilled water 2 times, 5 minutes each, at room temperature and dried on Whatmann paper, specimen side up. Grids were then analyzed on an H-7600 Hitachi Transmission Electron Microscope (Tokyo, Japan). NIH ImageJ software was used to mark the boundaries of lipid rafts that were imaged. The longest distance on the boundary of the captured and detected rafts, including the counter stained part, was used to determine the Ferret's diameter.

Cholesterol Depletion. Cholesterol was depleted from cell-free lipid rafts (lipid rafts previously isolated from cells) by treatment with 10 mMol/L of methyl-beta-cyclodextrin (MβCD) (Sigma-Aldhrich Company, St Louis, MO, USA) for 18-24 hours at 4°C before their use in the raft ELISA according to previously published report [23]. YH16.33 and A20 co-cultured cells, with or without chicken ovalbumin antigen were treated with 10 mM MβCD for 15 minutes at 37°C as per earlier published report [50] and isolated lipid rafts were examined by transmission electron microscopy.

## Results

### Characterization of detergent-free rafts from a CD4^+ ^T cell line

**Figure 1:**
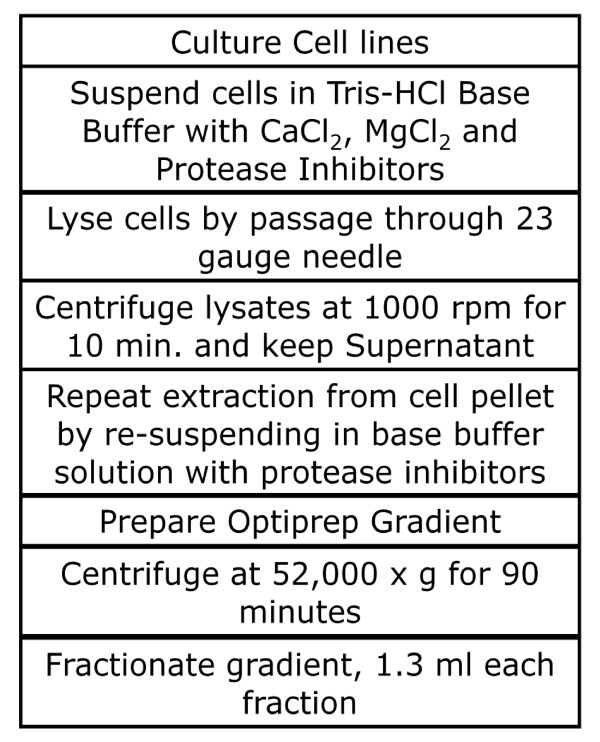
**The detergent-free isolation protocol of lipid rafts**. Six × 10^7 ^YH16.33 cells were centrifuged at 250 g for 10 minutes and the pellet was re-suspended in a base buffer consisting of 20 mM Tris-HCl, 250 mM Sucrose(pH 7.8), supplemented with 1 mM CaCl_2 _and 1 mM MgCl_2 _. The cells were then lysed by passing through a 23 gauge needle 20 times. The lysates were centrifuged at 1000 rpm for 10 minutes and the supernatant was saved. The pellet was re-suspended in fresh base buffer and then again passed though a 23 gauge needle 20 times. After centrifugation at 1000 rpm for 10 minutes again at 4°C, the supernatant was collected and pooled with the previously collected supernatant. An OptiPrep (Sigma-Aldrich, St. Louis, MO) gradient was prepared with a final 25% OptiPrep dilution at the bottom of an ultracentrifuge tube and overlaid with 20%, 15%, 10%, 5% and 0% OptiPrep solutions. The gradient was centrifuged at 52,000 × g for 90 minutes at 4°C and nine 1.3 ml aliquots from the top of gradient were collected and stored at -20°C until further analysis.

**Figure 2:**
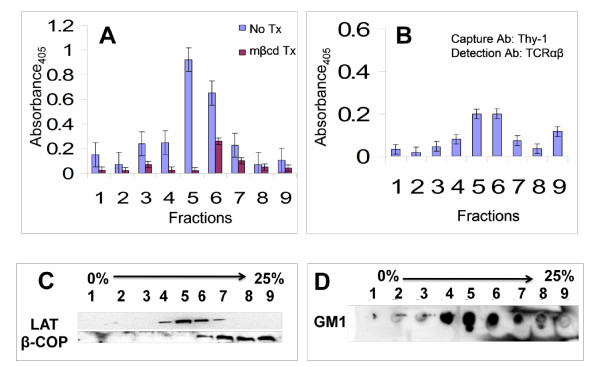
**Analysis of detergent-free isolated lipid rafts by raft ELISA and SDS-PAGE**. Anti-Thy-1, which recognizes a GPI-anchored protein known to be a raft constituent, Thy-1, was used as the capture antibody that coated the microtitre wells. Wells were incubated with the detergent-free fractions isolated from YH16.33 cells pretreated (Tx) with MβCD (A), or left untreated (No Tx) (A & B). Captured rafts were detected by biotinylated anti-Ly-6 antibody (A) or anti-TCRαβ antibody (B), followed by avidin-HRP. The assay was developed by adding ABTS peroxidase substrate and the absorbance was read by a Spectramax 190 plate reader. Fractions were also analyzed by running SDS-PAGE on 4-20% Tris-HCl gels followed by the transfer of proteins onto a PVDF membrane. Membranes were probed with anti-LAT or anti-β-COP (C), followed by the appropriate secondary antibodies. GM1 was detected in these fractions by dot-blot on PVDF membrane with biotin-cholera toxin β and avidin-HRP conjugate (D). Blots were observed using a chemiluminescence kit (Pierce, Rockford, IL) as described in the Materials and Methods section. Shown is a representation of at least three independent experiments.

Detergents promote coalescence of lipid rafts that may undermine assessment of raft heterogeneity [51,52]. To overcome the problems that are associated with the use of detergent in isolating lipid rafts from plasma membrane, we chose to isolate and characterize lipid rafts from a T-T hybrid CD4^+ ^T cell line in the absence of a non-ionic detergent. To achieve this, we adopted an isolation procedure used for detergent-free lipid rafts from an epithelial cell line [46], as shown in Figure 1. To assess the success of the isolation procedure and identify which density gradient fractions were enriched in lipid rafts from YH16.33, a T-T hybrid cell line, following detergent-free isolation, we carried out raft-ELISA using monoclonal antibodies directed to Thy-1 and Ly-6A.2, two GPI-anchored proteins known to localize in lipid rafts [14,15,23]. Anti-Thy-1 mAb was coated on the ELISA plate and used to capture detergent-free lipid rafts and biotinylated anti-Ly-6A.2 followed by avidin-HRP was used for detection. Figure 2A shows that fractions 5 and 6 contained lipid rafts. Cholesterol is an essential component of lipid rafts and thus, cholesterol-depleting compounds destabilize these membrane structures [50]. To assess the specificity of the captured membrane rafts, we treated the cell-free fractions with such a compound, methyl-beta-cyclodextrin (MβCD), at 10 mM for 18-24 hours. Incubation of lipid raft fractions with MβCD significantly decreased the capture and detection of Thy-1 and Ly-6 lipid raft subsets (Figure 2A). Through our binary approach of capture and detection of rafts we observed the presence of the antigen receptor, TCRαβ, in fraction numbers 5 and 6, as well (Figure 2B). Enrichment of TCRαβ in rafts has been observed by other investigators [53,54]. However, TCRαβ is present in the heavy fraction (fraction 9) which perhaps reflects its representation in the non-raft fractions as previously reported [29]. Alternately, the presence of TCRαβ in the heavy fraction reflects its presence in the cellular organelles (ER/Golgi etc), which is expected. To further analyze these fractions we carried out SDS-PAGE followed by Western blot analysis to detect Linker of activated T cells, LAT, another known component of lipid rafts [55] (Figure 2C). Ganglioside (GM1), another lipid raft marker, was detected in fractions 4-6 when dot blots of isolated detergent-free fractions were probed with HRP-conjugated Cholera toxin-β chain (Figure 2D). In contrast, β-COP, a Golgi-resident protein and a non-raft marker representing an internal cellular compartment was absent from these fractions (Figure 2C). Taken together, the raft ELISA and biochemical data show that detergent-free lipid rafts are present in fractions 4, 5 and 6 of the density gradient.

### Visualization and determination of size of lipid rafts using electron microscopy

**Figure 3:**
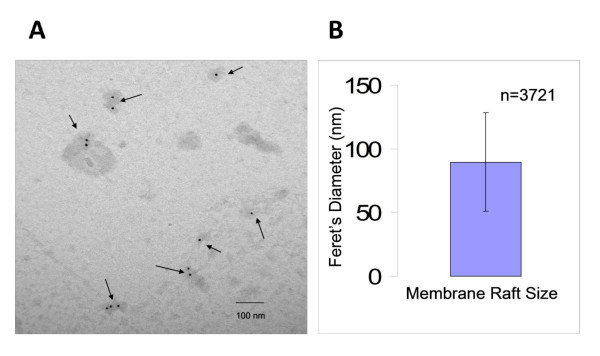
**Size of lipid rafts isolated from YH16.33 cells by the detergent-free isolation method**. Lipid rafts from the YH16.33 T cell line were captured and detected with anti-Thy-1 and anti-Ly-6 antibodies, respectively, on formvar coated nickel grids. A representative micrograph of YH16.33 lipid rafts (as shown with arrows) from fractions 5 (A) is shown. The average Feret's diameter of lipid rafts collected from fractions 5 is shown (B). Error bars show average size (nm) +/- standard deviation. Each micrograph was at a 40,000 × magnification. The micrograph is representative of at least three sets of experiments and the quantitative data is derived from all the experiments (n = 3721 lipid rafts).

Size of lipid rafts, reported in the literature using a variety of biophysical methods, has ranged from 10-100 nm in diameter [4,56,57]. Isolating the rafts from the plasma membrane in the absence of detergents and assessing their size will confirm their physical presence on the plasma membrane and will help clarify the disconnect between the biochemical and biophysical methods used to study these membrane entities. To examine the heterogeneity in size we used a clonal cell line, YH16.33, to generate detergent-free lipid rafts. Cell-free rafts from fraction 5 of the gradient were captured on nickel grids with anti-Thy-1 mAb and analyzed for the presence of Ly-6A.2 protein, another raft marker, with an anti-Ly-6A.2 mAb conjugated to biotin followed by anti-biotin antibody conjugated to 10 nm colloidal gold (see Materials and Methods section). Captured and detected rafts averaged 89.7 +/- 38.8 nm (n = 3721) in diameter (Figure 3A &3B). Isolated lipid rafts are those structures that were both immune-stained (i.e. those containing gold particles) and showed counter staining with Tannic acid and Uranyl acetate, which stains lipids. These results highlight the innate heterogeneity of the size of rafts on the plasma membrane of a clonal cell line.

### Lipid rafts coalesce in the presence of antigen presenting cells

**Figure 4:**
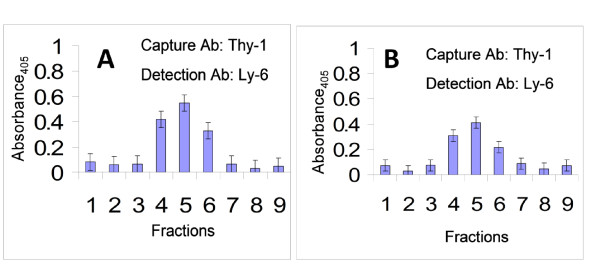
**Analysis of detergent-free lipid rafts by Raft ELISA**. Lipid rafts were isolated from T cell - APC co-cultures. YH16.33 cells were co-cultured with A20 cells in the absence (A) or presence (B) of 1 mg/ml chicken ovalbumin (specific antigen) for 18-24 hours and lipid rafts were isolated using the detergent-free density gradient method. Each density gradient was analyzed by Raft ELISA by capturing with anti-Thy-1 and detecting with biotinylated anti-Ly-6A.2 followed by avidin-HRP. A representative analysis of at least 3 independent experiments is shown.

**Figure 5:**
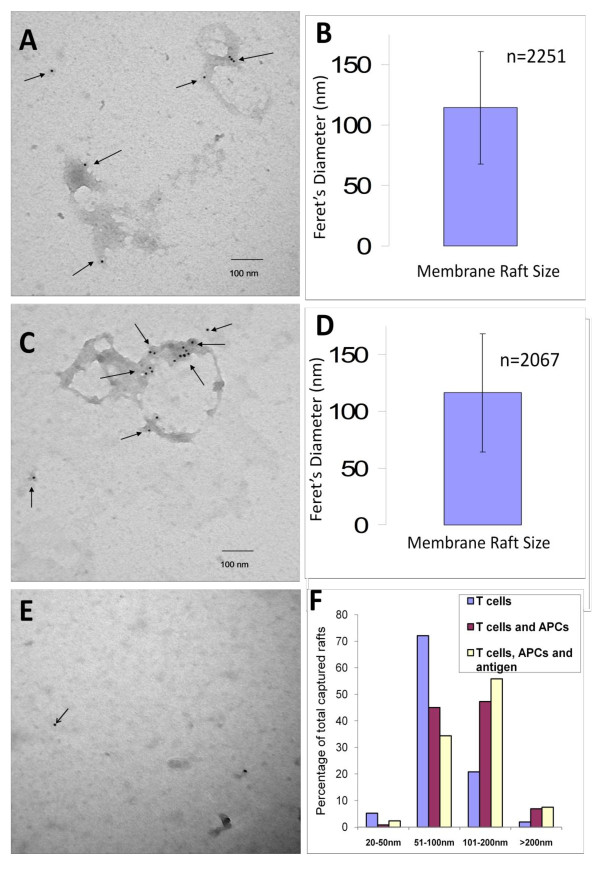
**Coalescence of lipid rafts isolated, detergent-free, from T cell - APC co-cultures**. YH16.33 cells were co-cultured with A20 cells in the absence (A & B) or presence (C & D) of 1 mg/ml chicken ovalbumin (specific antigen) for 18-24 hours and lipid rafts were isolated using the detergent-free density gradient method. Detergent-free lipid rafts in fraction 5 from YH16.33 + A20 (A), YH16.33 + A20 + chicken Ovalbumin (C) or A20 alone (E) were captured with anti-Thy-1 on formvar coated nickel grids and detected with biotinylated anti-Ly-6A.2 followed by anti-biotin conjugated to 10 nm colloidal gold and visualized with a Hitachi H-7600 transmission electron microscope. The average Feret's diameter of lipid rafts collected from fractions 5 of YH16.33 and A20 co-cultures either in the absence (B) or the presence (D) is shown. Error bars show average size (nm) +/- standard deviation. Lipid rafts from each group (T cells alone, T cells + APCs, T cells + APCs + antigen) were sized using NIH ImageJ software and their size distribution shown in nanometers is shown (F). Each micrograph was at a 40,000 × magnification. The micrograph shown are representative of at least three sets of experiments and the quantitative data is derived from all the experiments (n = 2251 lipid rafts for YH16.33+A20 and n = 2071 lipid rafts for YH16.33+A20+antigen groups).

**Figure 6:**
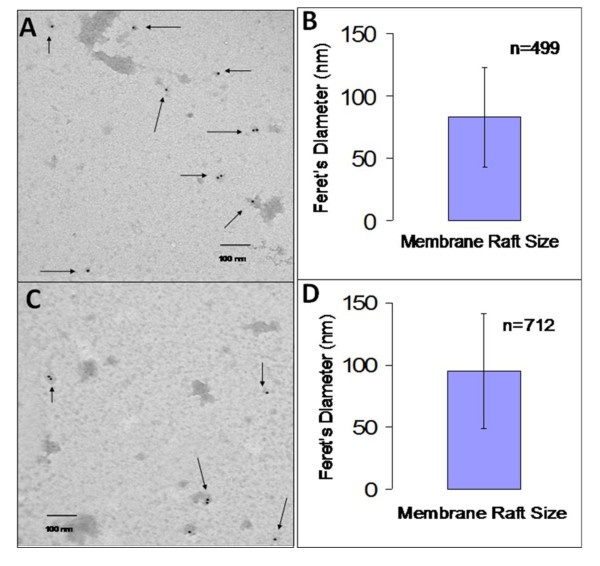
**Lipid raft coalescence is cholesterol-dependent**. Lipid rafts were isolated from YH16.33 cells co-cultured with A20 cells in the absence (A & B) and presence (C & D) of chicken ovalbumin after the treatment of co-cultured cells with MβCD for 15 minutes. Lipid rafts from fraction 5 of the density gradient were captured with anti-Thy-1 on formvar coated nickel grids and detected with biotinylated anti-Ly-6A.2 followed by anti-biotin colloidal gold conjugate. A representative micrograph (3 independent experiments) of lipid rafts (shown by arrows) from MβCD treated YH16.33 cells co-cultured with A20 cells in the absence (A) and presence (C) of antigen is shown. The average Feret's diameter of lipid rafts generated from YH16.33 and A20 co-cultures in the absence (C) and presence of antigen (D) is shown. Each micrograph was 40,000 × magnification.

We next sought to examine alterations in the lipid raft size and structure in CD4^+ ^T cell after exposure to a specific antigen. Lipid rafts are known to contribute to the formation of the immunological synapse which is considered to be a large coalesced raft formed at the contact site of a CD4^+ ^T cell and antigen presenting cell during T cell activation [4,14,27]. Previous studies have also suggested that lipid rafts take on the role of an anchoring platform for a number of signaling proteins [12-14]. To examine changes in the size of lipid rafts on the plasma membrane of T cells after engagement of their signaling receptors, we incubated YH16.33 T cells, with the antigen presenting cell, A20, in the presence and absence of a specific antigen, chicken ovalbumin. For each culture, detergent-free lipid rafts were isolated on an OptiPrep gradient after ultracentrifugation and lipid raft fractions were identified by raft ELISA (Without antigen, Figure 4A and with antigen, Figure 4B). Lipid rafts from fraction 5 were captured and detected with anti-Thy-1 and anti-Ly-6A.2 antibodies, respectively, for visualization under the electron microscope. Figure 5 shows that both in the absence (Figure 5A) and the presence (Figure 5C) of antigen, we frequently observed larger membrane entities (> 500 nm) composed of rafts attached to one another, and thus, appeared coalesced. These qualitatively distinct coalesced large rafts were not observed in the raft preparations from YH16.33 T cells alone. Moreover, the capture and detection of lipid rafts was T cell specific since we were unable to capture and detect lipid rafts generated from APCs (A20 cell line) alone with anti-T cell specific antibodies (Figure 5E). The average diameter of the rafts captured from T cells co-cultured with APCs in the absence of antigen was 116.327 +/- 52.112 nm (n = 2251) (Figure 5B). The average diameter of the rafts captured from T cells cultured with APCs in the presence of antigen was 114.430 +/- 46.748 nm (n = 2067) (Figure 5D). The diameter of both cultures of T cells with APCs, with or without antigen, produced similar sizes, although both were larger than un-stimulated YH16.33 cells (89.7+/-38.8 nm). About 72% of the rafts isolated, from the T cells in the absence of interaction with APC, ranged between 50-100 nm, and 22% ranged from 101-200 nm (Figure 5F). In contrast, the rafts isolated from YH16.33 with APC, either in the presence or the absence of antigen showed a shift towards higher size, with 38%-46% of total rafts showing 50-100 nm size distribution and 49% - 57% of 101-200 nm size (Figure 5F). When the co-cultures were treated with MβCD prior to lipid raft isolation there was a noticeable depletion in the formation of a circular shape of membrane rafts in YH16.33 and A20 co-cultures both in the absence (Figure 6A) and presence of antigen (Figure 6C). The average diameter of lipid rafts isolated from the co-cultures with and without antigen treated with MβCD was 83 +/- 39.9 nm (n = 499) and 95.2+/- 46.2 nm (n = 712), respectively (Figure 6B &6D). Taken together, our data suggests that prior to co-culture with APCs the average size of the lipid rafts are relatively small (89.7+/- 38.8 nm) and that rafts coalesce on the plasma membrane of CD4^+ ^T cells as they interact with APCs even in the absence of an antigen.

### Analysis of detergent-extracted lipid rafts

**Figure 7:**
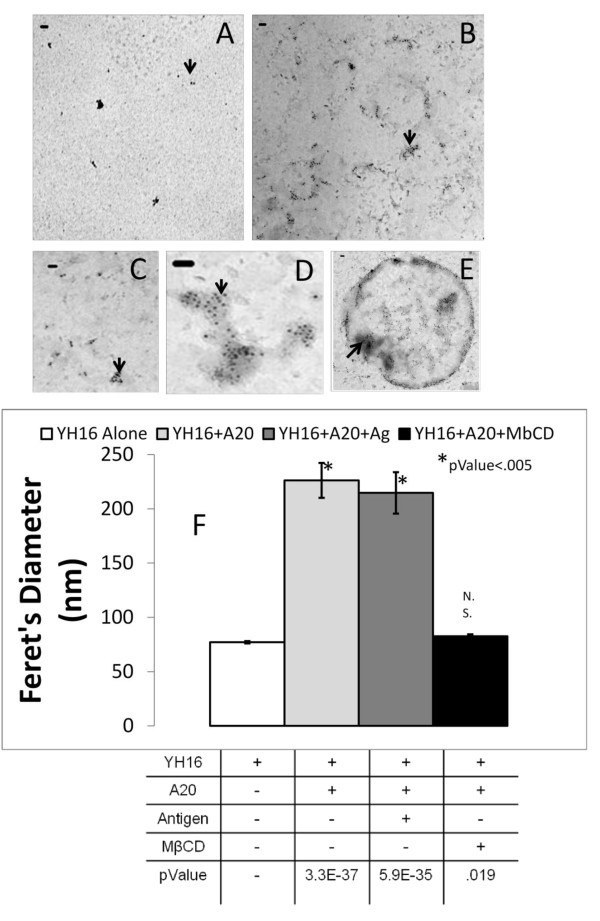
**Analysis of detergent-resistant (isolated using 1% Triton X-100) lipid rafts**. Lipid rafts were isolated from un-stimulated YH16.33 T cells (B), and YH16.33 cells co-cultured with A20 cells in the absence of antigen (C-E). Rafts were captured with anti-Thy-1 on the nickel grids followed by their detection with biotinylated anti-Ly-6A.2 and anti-biotin colloidal gold. Various sizes of lipid rafts ranging from less than 100 nm to several μm were visualized (C-E) and quantified (F). Feret's diameter of lipid rafts was determined for rafts isolated from YH16.33 alone (open squares), YH16.33 with A20 cells in the absence of antigen (light square), YH16.33 with A20 in the presence of antigen (grey square) and YH16.33 with A20 exposed to MβCD (black square) are shown. Asterisk (*) indicates significant differences and n.s denotes not significant differences from the YH16.33 alone group (F). Absence of lipid rafts isolated from A20 cells alone using anti-Thy-1 and anti-Ly-6A.2 antibodies to capture and detect, respectively is shown in A (negative control). Three independent experiments were carried out and 5 photographs were taken at 5 distinct regions on each grid. bar = 100 nm.

Use of non-ionic detergents for the extraction of lipid rafts from cells in a variety of cells has been controversial [51,52]. It has been suggested that these detergents promote coalescence of lipid rafts that may undermine assessment of raft heterogeneity. To address this issue, we wanted to compare detergent isolated rafts with those isolated using the detergent-free methodology proposed by McDonald and Pike [46]. We compared the size of lipid rafts from YH16.33 and A20 co-cultures that were isolated in the presence of the detergent, Triton X-100 (1% concentration) and examined their size by EM. These rafts were captured with an anti-Thy-1 mAb and detected with an anti-Ly-6A.2 antibody. Similar to the detergent-free rafts, lipid rafts isolated from YH16.33 alone with detergent-based isolation protocols yielded membrane entities less than 100 nm in diameter (Figure 7B &7F), as reported before [23]. In contrast to rafts isolated in a detergent-free environment, lipid rafts isolated in the presence of detergent from co-cultures of YH16.33 and A20 cells in presence or absence of specific antigen showed a higher frequency of macrodomains, some of which were up to micrometers in size (Figure 7C-E). However, like in the detergent-free case, frequency of macrodomain formation did not depend on the presence of antigen (Figure 7F). To examine if the presence of cholesterol was critical for formation of these large membrane domains we exposed the YH16.33 and A20 co-cultures to MβCD for 15 minutes prior to detergent isolation of lipid rafts. The isolated lipid rafts were captured and detected with anti-Thy-1 and anti-Ly-6A.2 monoclonal antibodies respectively. MβCD treated cultures showed considerably lower numbers of macrodomains than the untreated cultures (Figure 7F). These data suggest that cholesterol is necessary for formation and/or stability of the macrodomains we observed. Taken together, these results indicate that detergent does not affect the average size of rafts during isolation. However, in the presence of APCs, rafts isolated from T cells by detergent-based methods are considerably larger than the detergent-free rafts, supporting, perhaps, the hypothesis of detergent-dependent coalescence of lipid rafts that has been reported by other investigators [55]. Regardless of the size of these detergent-resistant domains their macro structure is dependent on the presence of cholesterol and independent of the antigen.

## Discussion

Heterogeneity of lipid rafts in the plasma membrane and their re-organization during ligand-receptor interactions plays an important role in cell signaling [14]. Resonance energy transfer (FRET) [15,56,58], super-resolution microscopy, [57,59] and other biophysical methods, have provided significant insights into establishing the existence and heterogeneity of these nano-size membrane domains. Analysis of lipid rafts, after immune-EM staining of intact plasma membrane, has also been useful in providing insights into the size and heterogeneity of lipid rafts [60]. Use of these methods in examining complex signaling cascades is challenging. Multitude of signaling proteins, participating in signal transduction, in native form or after post-translational modifications (phosphorylation) requires their visual detection simultaneously. Development of sensors allowing detection of several signaling molecules is currently underway. In here, we have examined alterations in size and composition of these membrane nano-domains following cellular interaction, on a single raft and raft-subpopulation basis. Use of biochemical approach to assess trafficking of native and post-translationally modified signaling receptors, moving in and out of lipid rafts isolated in the absence of detergent will be robust and without confounding issues with the use of detergents. Deciphering changes in size and composition in the same set of immune-isolated lipid raft populations is critical. While the biochemical approaches for examining the role of lipid rafts in spatiotemporal signaling in CD4^+ ^T cells can be remarkably robust, in as much as it has potential for analysis of a complex series of a multitude interacting molecules in the signal transduction cascade. However, this reductionist approach has inherent limitations and needs to be complimented by dynamic cell imaging showing interactions of multitude signaling proteins on the plasma membrane. The biochemical approaches using detergent-free lipid rafts, as well as the biophysical/dynamic cell imaging approaches currently underway are essential for developing a thorough understanding of spatial and temporal regulation of cell signaling.

The data presented here suggest that antigen and its recognition by TCRαβ are not the primary mechanism for the creation of macrodomains on the membrane, since we find them to be formed in the absence of specific antigen recognition. It is long been recognized that T cells interact with antigen-presenting cells in two phases. The first step requires nonspecific adhesion involving interactions between a β1 integrin, LFA-1 on T cells with the ligand, ICAM-1, expressed on antigen presenting cells [61]. In the second phase, the antigen receptor senses the antigen presented by the APC. The initial nonspecific interactions help launch the second phase, where the antigen receptor (TCRαβ) senses an antigen presented by the antigen-presenting cells. Detachment of T cells from APC occurs in the absence of recognition of an antigen. This opens up the opportunity to bind and sense the antigen on another APC. The data presented here suggest that during the first set of interactions between CD4^+ ^T cells and APC the lipid rafts on T cells are spatially organized and coalesce. Previous reports have described antigen-independent immunological synapses between naïve CD4^+ ^T cells and dendritic cells [62]. Functional consequence of the antigen-independent interaction range from tyrosine phosphorylation, little calcium response and survival signals. It appears that these interactions allow survival of naïve T cell in vivo. However, the relationship between the antigen-independent synapse formation and coalescence of lipid rafts during T cell APC interactions needs to be elucidated. Further investigation needs to be carried out to understand the mechanism, and functional importance of this early spatial reorganization of the plasma membrane. Extent of raft coalescence and molecules that accumulate in it may depend on the source of interacting CD4^+ ^T cell and degree of ligation of the antigen receptor and co-receptor [63]. In addition, a functional role of lipid rafts may not be the same in distinct subsets of differentiated CD4^+ ^T cells. For example, activated Th1 and Th2 cells behave differently in their re-organization of lipid rafts. While the antigen receptor is easily recruited in the lipid rafts in Th1 cells, similar recruitment is not observed in activated Th2 cells [64]. Furthermore, it will be crucial to ascertain whether this re-organization reflects the underlying properties of the nanoscale assemblies that show additional interconnections when CD4^+ ^T cells interact with antigen-presenting cells as suggested by a recent report [65]. While antibodies to T cell surface proteins were used in our experiments to capture and detect isolated lipid rafts, it is possible that the captured coalesced rafts have some membranes belonging to APC. We have not directly tested this idea. Future experiments, where antibodies directed against MHC class II proteins and anti-TCRαβ used to capture and detect coalesced lipid rafts will be able to address this issue.

## Conclusions

We conclude that lipid rafts on CD4^+ ^T cell membranes coalesce to form larger structures, after interacting with antigen presenting cells even in the absence of a foreign antigen. Findings presented here indicate that lipid raft coalescence occurs during cellular interactions prior to sensing a foreign antigen.

## List of Abbreviations

CD4: cluster differentiation antigen 4; APC: antigen presenting cells; MβCD: methyl-beta cyclodextran; GPI: glycosylphosphatidyl-inositol; TCR: T cell receptor

## Competing interests

The authors declare that they have no competing interests.

## Authors' contributions

CK and MDN performed experiments, analyzed the data and partly wrote the manuscript, AKB designed the project, analyzed the data, wrote the manuscript and edited the manuscript. All authors have read and approved the final manuscript.
